# Donor-derived cell-free DNA as a noninvasive biomarker for diagnosis and monitoring of acute rejection after liver transplantation

**DOI:** 10.3389/fimmu.2026.1769538

**Published:** 2026-05-11

**Authors:** Zhigao Deng, Meicheng Yang, Quanwei Cheng, Zhongshan Lu, Qifa Ye, Yan Xiong, Shaojun Ye

**Affiliations:** 1 Zhongnan Hospital of Wuhan University, Institute of Hepatobiliary Diseases of Wuhan University, Transplant Center of Wuhan University, National Quality Control Center for Donated Organ Procurement, Hubei Key Laboratory of Medical Technology on Transplantation, Hubei Provincial Clinical Research Center for Natural Polymer Biological Liver, Wuhan, Hubei, China; 2 NHC Key Laboratory of Translational Research on Transplantation Medicine, The 3rd Xiangya Hospital of Central South University, Changsha, China

**Keywords:** acute rejection, biomarker, donor-derived cell-free DNA, liver transplantation, receiver operating characteristic

## Abstract

**Background and aims:**

Acute rejection (AR) is a common complication after liver transplantation. Current diagnostic modalities for acute rejection are either invasive or lack sufficient sensitivity. Therefore, the present study aimed to develop a novel and sensitive diagnostic tool for predicting AR after liver transplantation. Specifically, we investigated whether serum donor-derived cell-free DNA (dd-cfDNA) is closely associated with the occurrence of post-transplant AR.

**Methods:**

A prospective single-center diagnostic study enrolled 40 primary whole liver transplant recipients, divided into an indicative biopsy cohort (abnormal liver function requiring biopsy) and a protocol biopsy cohort (stable/mildly abnormal liver function without biopsy indication). The dd-cfDNA levels (absolute copy number and relative quantification) were dynamically monitored 14 days to 1 year post-transplant. Using pathological biopsy as the gold standard, receiver operating characteristic (ROC) curve analysis and logistic regression models compared the diagnostic efficacy of dd-cfDNA and liver function indices for AR.

**Results:**

Our study demonstrated that circulating dd-cfDNA levels were significantly elevated in liver transplant recipients with acute rejection (AR) compared to those without. The diagnostic cutoffs were determined as dd-cfDNA% ≥10.39% (sensitivity: 95%, specificity: 90%) and dd-cfDNA ≥1928 copies/mL (sensitivity: 70%, specificity: 90%). The area under the curve (AUC) for dd-cfDNA% was 0.940, and for dd-cfDNA was 0.823, both outperforming routine liver function tests. After effective anti-rejection therapy, dd-cfDNA levels rapidly decreased, correlating with clinical improvement, as well as improvements in liver function and histopathology.

**Conclusions:**

As a sensitive and non-invasive biomarker, dd-cfDNA can effectively predict the occurrence of AR after liver transplantation. Moreover, changes in dd-cfDNA levels facilitate the assessment of therapeutic efficacy, providing crucial references for optimizing immunosuppressive regimens and improving patient outcomes.

## Graphical Abstract

**Figure:**
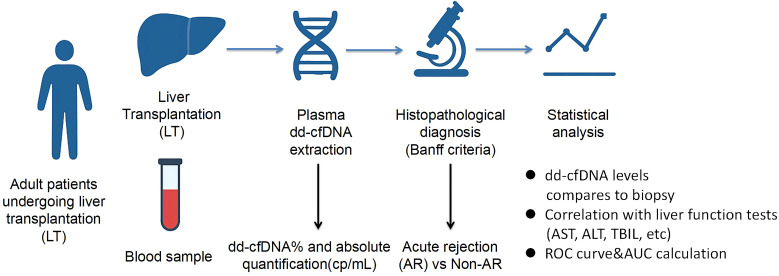
Study design for assessing plasma donor-derived cell-free DNA as a biomarker of acute rejection after liver transplantation.

## 1 Introduction

Liver transplantation (LT) remains the definitive and most effective treatment for patients with end-stage liver disease (1, 2). Over the past few decades, continuous advances in surgical techniques, perioperative management, and immunosuppressive strategies have significantly improved both graft and patient survival (3, 4). However, immune-mediated graft rejection continues to represent a major cause of long-term graft dysfunction and remains a critical determinant of patient outcomes (5). Among post-transplant immune complications, acute rejection (AR) is relatively common, typically occurring within the first year after LT, with an incidence of approximately 20–30% (6). Although the liver exhibits a unique degree of immunologic tolerance compared with other solid organs, AR can still result in substantial hepatocellular injury, and, if not promptly recognized and treated, may progress to chronic rejection or graft loss (7, 8).

Traditionally, the diagnosis of AR has relied on liver biopsy and histopathological evaluation, which are considered the gold standard. However, biopsy is invasive, costly, and subject to sampling error and interobserver variability. Furthermore, it cannot provide continuous or early detection of immune-mediated graft injury (9). Conventional biochemical indicators such as alanine aminotransferase (ALT), aspartate aminotransferase (AST), and total bilirubin (TBIL) are routinely used to monitor graft function but lack specificity and sensitivity for distinguishing rejection from other causes of dysfunction (10, 11). These limitations underscore the urgent need for a noninvasive, sensitive, and quantitative biomarker that can accurately reflect immune-mediated allograft injury in real time.

In recent years, the advent of molecular diagnostic techniques has introduced donor-derived cell-free DNA (dd-cfDNA) as a promising biomarker for noninvasive transplant monitoring (12, 13). Donor-derived cell-free DNA consists of short DNA fragments released into the recipient’s plasma from apoptotic or necrotic donor cells and exhibits a very short circulating half-life of approximately 30 minutes to 2 hours (14, 15). Under normal graft conditions, dd-cfDNA levels remain low because cell turnover is limited. During rejection, activated recipient immune cells can injure multiple donor-derived liver cell populations, including hepatocytes, cholangiocytes/biliary epithelial cells, and endothelial cells such as sinusoidal and venous endothelial cells, thereby contributing to an increase in circulating dd-cfDNA (16). These fragments can be sensitively detected and quantified through next-generation sequencing (NGS) or digital droplet PCR (ddPCR), which distinguish donor and recipient DNA based on single-nucleotide polymorphisms (SNPs) or insertion/deletion polymorphisms (17, 18). The dd-cfDNA can be expressed either as an absolute concentration (copies/mL) or as a relative fraction (% of total cfDNA). Previous studies have shown that the relative fraction (dd-cfDNA%) may better reflect graft-specific injury because it corrects for fluctuations in total cfDNA caused by factors such as infection, inflammation, or surgical stress (19, 20).

The dd-cfDNA, representing fragments of DNA released from the injured donor-derived cells into the recipient’s circulation, has emerged as a promising non-invasive biomarker of allograft injury (21). In solid organ transplantation, particularly in kidney, heart, and lung transplants, numerous studies have demonstrated that elevated dd-cfDNA levels correlate strongly with biopsy-proven acute rejection and may even precede clinical manifestations or biochemical abnormalities (22–24). More recently, growing evidence suggests that dd-cfDNA may also serve as an early and dynamic indicator of immune-mediated graft injury following liver transplantation (25). However, the diagnostic thresholds, sensitivity, specificity, and predictive value of dd-cfDNA for detecting AR in liver transplant recipients remain incompletely defined, and the clinical utility of dd-cfDNA in this context warrants further investigation.

Therefore, this study aims to evaluate the diagnostic value of dd-cfDNA in identifying acute rejection among liver transplant recipients. By comparing dd-cfDNA levels between patients with and without biopsy-proven AR and analyzing their correlation with clinical parameters, we seek to determine the optimal cutoff values and assess the sensitivity, specificity, and predictive performance of dd-cfDNA as a non-invasive biomarker for early detection of acute rejection after liver transplantation.

## 2 Materials and methods

### 2.1 Study design and patient enrollment

This prospective, single-center study included 40 primary liver transplant recipients (whole grafts), who were followed from 14 days to 1 year post-transplantation. Participants were stratified into two cohorts according to biopsy pathway: an indicative (for-cause) biopsy cohort, comprising recipients who underwent liver biopsy because of abnormal liver function tests exceeding 2 times the upper limit of normal (ULN), with biopsy performed in the context of clinically suspected graft dysfunction, and a protocol biopsy cohort, comprising recipients with stable liver function or only mildly abnormal liver function (≤2 times the ULN) who underwent scheduled surveillance biopsies at 1, 6, and 12 months after transplantation. Histopathological examination of liver biopsy specimens was used as the reference standard for the diagnosis of acute rejection. Histopathological examination of liver biopsy specimens was used as the reference standard for the diagnosis of AR.

### 2.2 Inclusion and exclusion criteria

Inclusion criteria: Adult patients (18–80 years old) who underwent their first liver transplantation (whole grafts) and had complete clinical and follow-up data.

Exclusion criteria: Patients with severe infections, recurrent malignancy, or major postoperative complications (e.g., massive bleeding, multiorgan failure), as well as those who withdrew from follow-up or provided insufficient samples, were excluded.

All participants provided written informed consent prior to enrollment, and the study protocol was approved by the institutional ethics committee.

### 2.3 Ethical considerations

This study was approved by the institutional review board (IRB) of Zhongnan Hospital of Wuhan University and conducted in accordance with the ethical principles outlined in the Declaration of Helsinki and relevant national regulations. Written informed consent was obtained from all participants before enrollment, ensuring that participants understood the purpose, procedures, potential risks, and benefits of the study. All patient data were anonymized and handled in accordance with privacy and confidentiality standards.

### 2.4 Sample collection

Peripheral blood samples (8–10 mL) were obtained from each liver transplant recipient at predefined time points of postoperative week 2 and months 1, 6, and 12. Samples were promptly centrifuged to isolate plasma, which was stored at -80 °C until further analysis for dd-cfDNA quantification. In the protocol biopsy cohort, liver biopsies were performed at predefined surveillance time points of 1, 6, and 12 months after transplantation. In the indicative (for-cause) biopsy cohort, liver biopsy was performed when acute rejection was suspected based on clinical presentation or abnormal liver function tests. For diagnostic analyses, dd-cfDNA samples were collected either on the day before biopsy or within a few hours before biopsy, and in all cases before liver biopsy and before the initiation of anti-rejection therapy.

### 2.5 The dd-cfDNA extraction and quantification using NGS

Plasma cfDNA was extracted using a commercial DNA extraction kit (QIAamp Circulating Nucleic Acid Kit, Qiagen) following the manufacturer’s instructions. NanoDrop was used only for routine assessment of extracted DNA purity, whereas dd-cfDNA quantification was performed using an SNP-based next-generation sequencing assay on the Illumina MiSeqDx platform.

Quantification of dd-cfDNA was performed using an AlloDx SNP-based next-generation sequencing assay on the Illumina MiSeqDx platform. The assay targeted 5,800 single-nucleotide polymorphism (SNP) loci for high-depth sequencing, enabling differentiation between donor- and recipient-derived cfDNA fragments. The absolute dd-cfDNA concentration was determined by normalizing donor-specific read counts to total cfDNA, while the relative dd-cfDNA fraction was calculated as the proportion of donor-derived cfDNA to total cfDNA within plasma samples.

### 2.6 Diagnosis and treatment of acute rejection

Acute rejection was diagnosed histologically using liver biopsy as the reference standard. All biopsy specimens were evaluated according to the Banff criteria for liver allograft rejection (26). Two independent, blinded pathologists reviewed each sample to minimize interobserver variability. Acute rejection severity was assessed using the Banff rejection activity index (RAI) score and classified as mild, moderate, or severe according to the extent of inflammatory infiltration and bile duct/venous endothelial injury.

For subclinical and mild acute rejection, close observation with a modest increase in tacrolimus dose is often sufficient, provided that drug levels are carefully monitored and follow-up liver biopsies are performed. Once histology confirms that rejection has improved or resolved, the tacrolimus dose should be promptly reduced to avoid toxicity. For moderate to severe acute rejection, intravenous methylprednisolone pulse therapy is typically given for 3 days at decreasing doses. In steroid-resistant severe rejection, lymphocyte-depleting agents such as antilymphocyte globulin (ALG), antithymocyte globulin (ATG), or anti-CD3 monoclonal antibodies may be used. In cases of irreversible rejection, re-transplantation should be considered.

### 2.7 Clinical data collection and analysis

Comprehensive clinical data were collected for each liver transplant recipient, including demographic information (age and sex), underlying liver disease, pre-transplant liver function, and donor liver ischemia times. At each follow-up, serial measurements of post-transplant liver function parameters, including ALT, AST, and TBIL, were recorded.

### 2.8 Statistical analysis

Statistical analyses were performed using SPSS version 26.0 (IBM Corp., Armonk, NY, USA) and GraphPad Prism version 9.0 (GraphPad Software, San Diego, CA, USA). Continuous variables were expressed as mean ± standard deviation (SD) or median (interquartile range, IQR), as appropriate, and categorical variables were presented as number (percentage). For baseline characteristics, between-group comparisons of continuous variables were performed using the Student’s t-test for normally distributed data or the Mann–Whitney U test for non-normally distributed data. Categorical variables were compared using the χ² or Fisher’s exact test. Paired *t*-test or Wilcoxon signed-rank test was used for pre- and post-treatment comparisons. Receiver operating characteristic (ROC) curves were generated to evaluate the diagnostic performance of dd-cfDNA, and the optimal cutoff value was determined using the Youden index. For ROC analysis, only one dd-cfDNA sample per patient was included. When multiple longitudinal samples were available, the single pre-biopsy, pre-treatment sample closest to the index biopsy time point was selected. A two-tailed *p* value < 0.05 was considered statistically significant.

## 3 Results

### 3.1 Baseline characteristics of the study cohort

A total of 40 liver transplant recipients were enrolled, including 20 patients with biopsy-proven acute rejection and 20 without histological evidence of rejection. Among the 20 biopsy-proven acute rejection cases, 8 (40.0%) were subclinical rejection identified on protocol biopsies, whereas 12 (60.0%) were clinically overt rejection diagnosed on indicative (for-cause) biopsies. Baseline demographic and clinical characteristics are summarized in **Table 1**. The mean age at transplantation was 42.9 ± 11.8 years (range, 21–67 years), and 33 recipients (82.5%) were male. There were no significant differences between the AR and non-AR groups with respect to age, sex, or BMI (all *p* > 0.05), indicating that the two groups were broadly comparable in terms of baseline demographic profile.

**Table 1:** Baseline characteristics of liver transplant recipients.

Characteristics	Total (n=40)	Rejection (n=20)	Non-Rejection (n=20)	*p value*
Gender (Male)	33 (82.5%)	15 (75.0%)	18 (90.0%)	0.223
Age (years)	42.9±11.8	46.5±10.2	39.3±12.5	0.053
BMI (kg/m^2^)	23.4±4.9	22.8±4.2	23.9±5.5	0.474
Post-transplant time (day)	49.7 ± 66.9	42.7 ± 72.7	56.8 + 61.6	0.512
Warm Ischemia Time (min)	11.8±2.1	12.4±2.1	11.1±1.9	0.048
Cold Ischemia Time (h)	6.4±0.7	6.6±0.7	6.3±0.7	0.184
Anhepatic Phase Time (min)	44.4±9.0	44.9±9.3	43.9±8.9	0.745
Surgical Method (Classic or Piggyback)	Classic 13 (32.5%) Piggyback 27 (67.5%)	Classic 4 (20.0%) Piggyback 16 (80.0%)	Classic 9 (45.0%) Piggyback 11 (55.0%)	0.096
Surgical Time (min)	308.7±64.4	299.0±65.0	318.9±64.0	0.341
Intraoperative Blood Loss (ml)	311.1±166.7	342.5±183.7	276.1±142.4	0.225
Transplant Indication				
HBC	28 (70.0%)	16 (80.0%)	12 (60.0%)	0.257
HCV	1 (2.5%)	0 (0%)	1 (5.0%)	0.331
Alcoholic Liver	2 (5.0%)	0 (0%)	2 (10.0%)	0.163
Autoimmune Liver	3 (7.5%)	2 (10.0%)	1 (5.0%)	0.591
Others	4 (10.0%)	2 (10.0%)	2 (10.0%)	0.042
ALT(U/L)	88.6±94.1	102.3±121.1	74.2±52.9	0.358
AST(U/L)	127.8±107.5	137.3±105.6	117.8±111.4	0.579
TBIL(umol/L)	261.3±203.1	265.5±197.2	257.0±214.4	0.899
Creatinine(umol/L)	104.6±98.7	110.9±103.7	98.1±95.5	0.690
MELD Score	36.4±11.0	36.8±11.0	36.1±11.4	0.847

Data are presented as mean ± SD or number (percentage). Continuous variables were compared using the Student’s *t*-test or the Mann–Whitney *U* test, and categorical variables using the χ² test or Fisher’s exact test. A *p* value < 0.05 was considered statistically significant; Abbreviations: BMI, body mass index; ALT, alanine aminotransferase; AST, aspartate aminotransferase; TBIL, total bilirubin; ALP, alkaline phosphatase; GGT, γ-glutamyl transferase; MELD, Model for End-Stage Liver Disease; HBC, hepatitis B–related cirrhosis; HCV, hepatitis C virus; AIH, autoimmune hepatitis; PSC, primary sclerosing cholangitis; PBC, primary biliary cholangitis.

Perioperative variables were also largely balanced. The only significant difference was observed in warm ischemia time, which was longer in the AR group than in the non-AR group (12.4 ± 2.1 vs. 11.1 ± 1.9 min, *p* = 0.048). This finding suggests that greater perioperative ischemic stress may have contributed to subsequent graft injury and increased susceptibility to acute rejection in this cohort. However, correlation analysis showed that warm ischemia time was not significantly associated with either fractional dd-cfDNA (*r* = 0.097, *p* = 0.551) or absolute dd-cfDNA (*r* = 0.043, *p* = 0.789) (**Supplementary Figure 1**). Cold ischemia time and anhepatic phase duration did not differ significantly between groups (both *p* > 0.05). Most recipients underwent the piggyback technique (67.5%), with a similar distribution of classic versus piggyback approaches in the AR and non-AR groups (*p* = 0.096). Surgical duration, intraoperative blood loss, and post-transplant sampling time were also comparable between groups (all *p* > 0.05). The finding that only a modest prolongation of warm ischemia time distinguished patients who subsequently developed AR suggests that perioperative ischemic injury may contribute to alloimmune activation and rejection risk in this cohort.

Regarding transplant indications, hepatitis B virus infection was the predominant cause, accounting for 70.0% of the overall cohort (80.0% in the AR group vs. 60.0% in the non-AR group, *p* = 0.257). Other etiologies included hepatitis C virus infection, alcoholic liver disease, autoimmune liver disease, and other causes, with distributions generally similar between groups (*p* values as shown in **Table 1**). Pre-transplant liver and renal function parameters (ALT, AST, TBIL, and creatinine) and MELD scores did not differ significantly between the AR and non-AR groups (all *p* > 0.05).

### 3.2 Histopathological confirmation and therapeutic response of acute rejection

**Figure 1:**
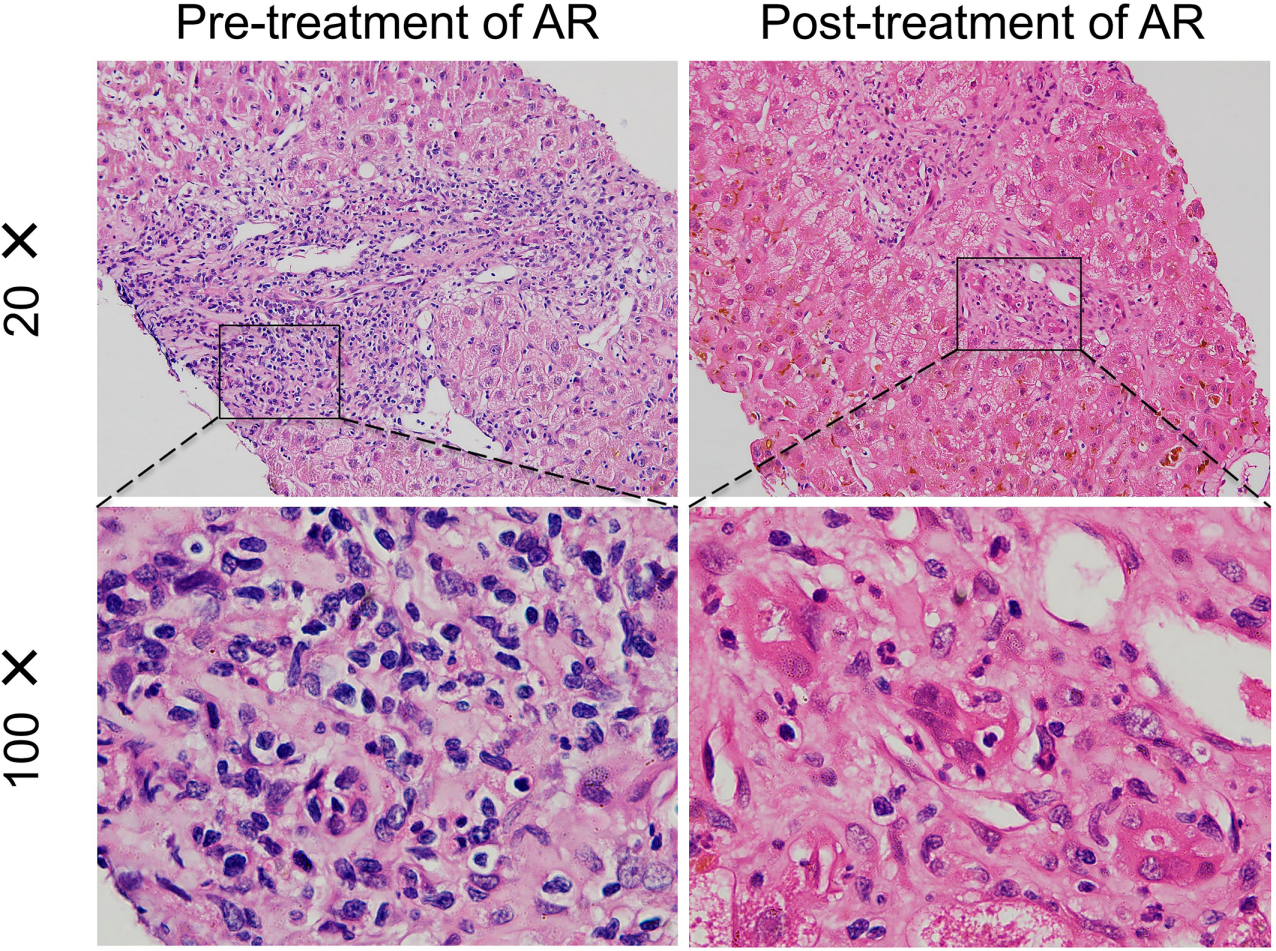
Histopathological findings of liver allografts before and after anti-rejection therapy. Representative liver biopsy images showing morphological changes in graft tissue before and after treatment of acute rejection.

Liver biopsy confirmed the diagnosis of acute rejection in patients with elevated dd-cfDNA levels (**Figure 1**). Before anti-rejection therapy, the liver allograft showed dense portal and lobular inflammatory infiltrates, predominantly composed of lymphocytes and eosinophils, accompanied by bile duct epithelial damage and hepatocellular necrosis, consistent with moderate to severe acute cellular rejection. Following effective anti-rejection treatment, the degree of portal inflammation and hepatocellular injury markedly decreased, with restoration of lobular architecture and resolution of bile duct injury.

### 3.3 Dynamic changes in dd-cfDNA and liver function parameters during acute rejection and after treatment

**Figure 2:**
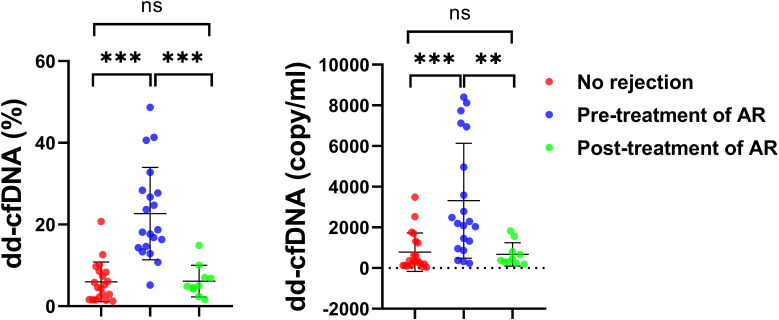
Dynamic changes in dd-cfDNA levels before and after anti-rejection therapy. Comparison of dd-cfDNA fraction (%) and absolute concentration (copies/mL) among the no-rejection, pre-treatment acute rejection (AR), and post-treatment AR groups. Statistical comparisons between the no-rejection group and the pre-treatment or post-treatment AR groups were performed using the Mann–Whitney U test. Comparisons between the pre-treatment and post-treatment AR groups were performed using the Wilcoxon signed-rank test. Levels of statistical significance are indicated as: **P < 0.01; ***P < 0.001; ns indicates no significant difference.

Consistent with the histological improvement observed after anti-rejection therapy, dd-cfDNA levels also decreased markedly after treatment. To assess the dynamic relationship between dd-cfDNA and graft injury, serial measurements of dd-cfDNA and conventional liver function parameters were analyzed in AR and non-AR recipients (**Figure 2**). At the time of biopsy-proven AR, both dd-cfDNA% and absolute dd-cfDNA levels were significantly elevated compared with those in non-rejection recipients (*p* < 0.001). The optimal diagnostic cutoffs identified for AR were dd-cfDNA% ≥ 10.39% (22.67 ± 11.31%) and dd-cfDNA ≥ 1928 copies/mL (3562.49 ± 2998.96 copies/mL). Following anti-rejection therapy, dd-cfDNA% and absolute dd-cfDNA levels decreased markedly (6.14 ± 3.88% and 671.83 ± 572.95 copies/mL, respectively; *p* < 0.001), approaching baseline values comparable to those observed in non-rejection recipients. Furthermore, dd-cfDNA levels tended to increase with the increasing histological severity of acute rejection (**Supplementary Figure 2**).

**Figure 3:**
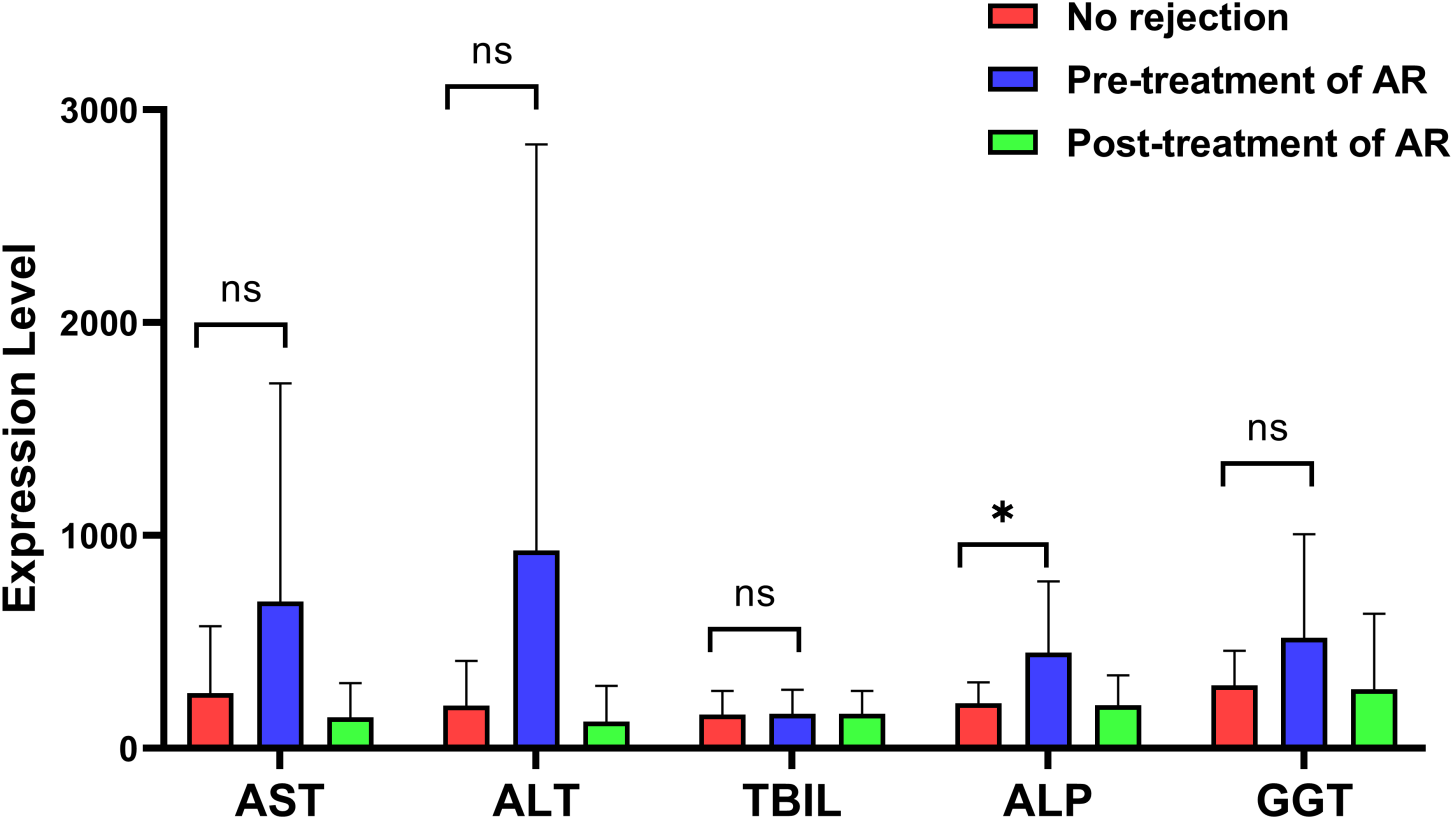
Liver function parameters among non-rejection, pre-treatment, and post-treatment groups. Comparison of conventional biochemical indicators, including AST, ALT, TBIL, ALP, and GGT, among the three clinical states. Statistical comparisons between the no-rejection group and the pre-treatment or post-treatment AR groups were performed using the Mann–Whitney U test. Comparisons between the pre-treatment and post-treatment AR groups were performed using the Wilcoxon signed-rank test. Levels of statistical significance are indicated as: *P < 0.05; ns indicates no significant difference.

In contrast, conventional liver function tests showed limited ability to distinguish AR from non-rejection (**Figure 3**). Serum AST, ALT, TBIL, and GGT levels tended to be higher in patients with AR before treatment but exhibited wide inter-individual variability and substantial overlap among the three groups, and most differences did not reach statistical significance. Only ALP showed a modest but significant elevation in the pre-treatment AR group compared with the other groups, and its levels did not normalize as completely as dd-cfDNA after therapy. Collectively, our results demonstrate the superiority of dd-cfDNA as a sensitive, organ-specific marker of graft injury compared to standard biochemical indices. While routine liver function tests lack the precision to isolate donor-specific damage due to their sensitivity to systemic variables, dd-cfDNA concentrations directly correlate with the degree of donor cell apoptosis and necrosis. Although the relative quantification (dd-cfDNA%) compensates for variations in total cfDNA, clinical interpretation must still account for non-rejection events, including severe ischemia–reperfusion injury and infection, which may independently influence these markers.

### 3.4 Diagnostic performance of dd-cfDNA compared with conventional liver function tests

**Figure 4:**
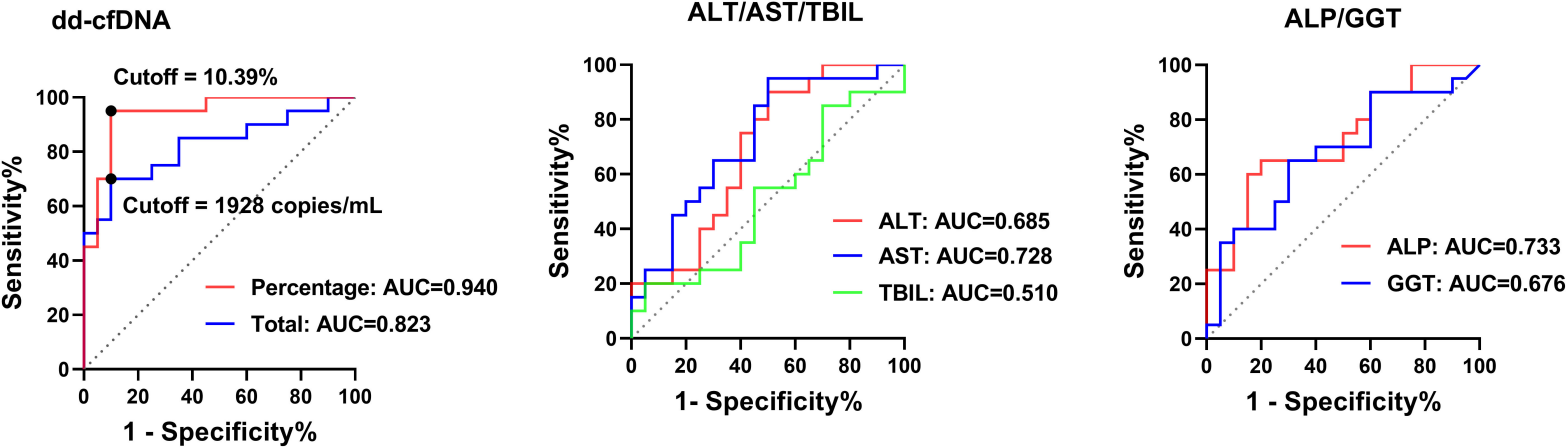
ROC curve analysis of dd-cfDNA and liver function tests for diagnosing acute rejection. Receiver operating characteristic (ROC) curves comparing the diagnostic performance of dd-cfDNA and conventional liver function markers in distinguishing acute rejection from stable graft function. The area under the curve (AUC) was calculated, and statistical significance was defined as *p* < 0.05.

The diagnostic accuracy of dd-cfDNA for biopsy-proven AR was evaluated by ROC curve analysis and compared with conventional liver function tests (**Figure 4**). dd-cfDNA% showed excellent discriminative ability, with an AUC of 0.94 (95% CI, 86.66-100.0; *p* < 0.001). Absolute dd-cfDNA also demonstrated good performance, with an AUC of 0.82 (95% CI, 68.87-95.63; *p* = 0.005). Using the optimal cutoffs derived from the ROC curves (dd-cfDNA%≥10.39% and dd-cfDNA≥1,928 copies/mL), dd-cfDNA% achieved a sensitivity of 95% and a specificity of 90%, whereas absolute dd-cfDNA yielded a sensitivity of 70% and a specificity of 90%.

By contrast, routine liver biochemistry provided only modest diagnostic utility. The AUCs were 0.69 for ALT (95% CI, 51.67–85.33; *p* = 0.045), 0.73 for AST (95% CI, 56.91–88.59; *p* = 0.013), 0.51 for TBIL (95% CI, 32.64–69.36; *p* = 0.913), 0.73 for ALP (95% CI, 57.66–88.84; *p* = 0.012), and 0.68 for GGT (95% CI, 50.69–84.56; *p* = 0.057) (**Table 2**). Several indices approached the performance of random classification, and none reached the level of accuracy observed for dd-cfDNA%, underscoring the superiority of dd-cfDNA as a diagnostic biomarker for AR. In an additional exploratory ROC analysis, combining fractional dd-cfDNA with AST slightly improved diagnostic performance for biopsy-proven acute rejection (AUC 0.950 vs. 0.940 for fractional dd-cfDNA alone), whereas the combination of absolute dd-cfDNA and AST showed only minimal improvement (AUC 0.827 vs. 0.823 for absolute dd-cfDNA alone) (**Supplementary Figure 3**).

**Table 2:** Diagnostic performance of dd-cfDNA and conventional liver injury biomarkers.

Biomarker	AUC	95% CI	*p* value
dd-cfDNA%	0.94	86.66-100.0	<0.001
Absolute dd-cfDNA	0.82	68.87-95.63	0.005
ALT	0.69	51.67-85.33	0.045
AST	0.73	56.91-88.59	0.013
TBIL	0.51	32.64-69.36	0.913
ALP	0.73	57.66-88.84	0.012
GGT	0.68	50.69-84.56	0.057

ROC analysis of dd-cfDNA and conventional liver function parameters for detecting acute rejection. AUC, area under the curve; CI, confidence interval; ALT, alanine transaminase; AST, aspartate transaminase; TBIL, total bilirubin; ALP, alkaline phosphatase; GGT, gamma-glutamyl transpeptidase.

**Figure 5:**
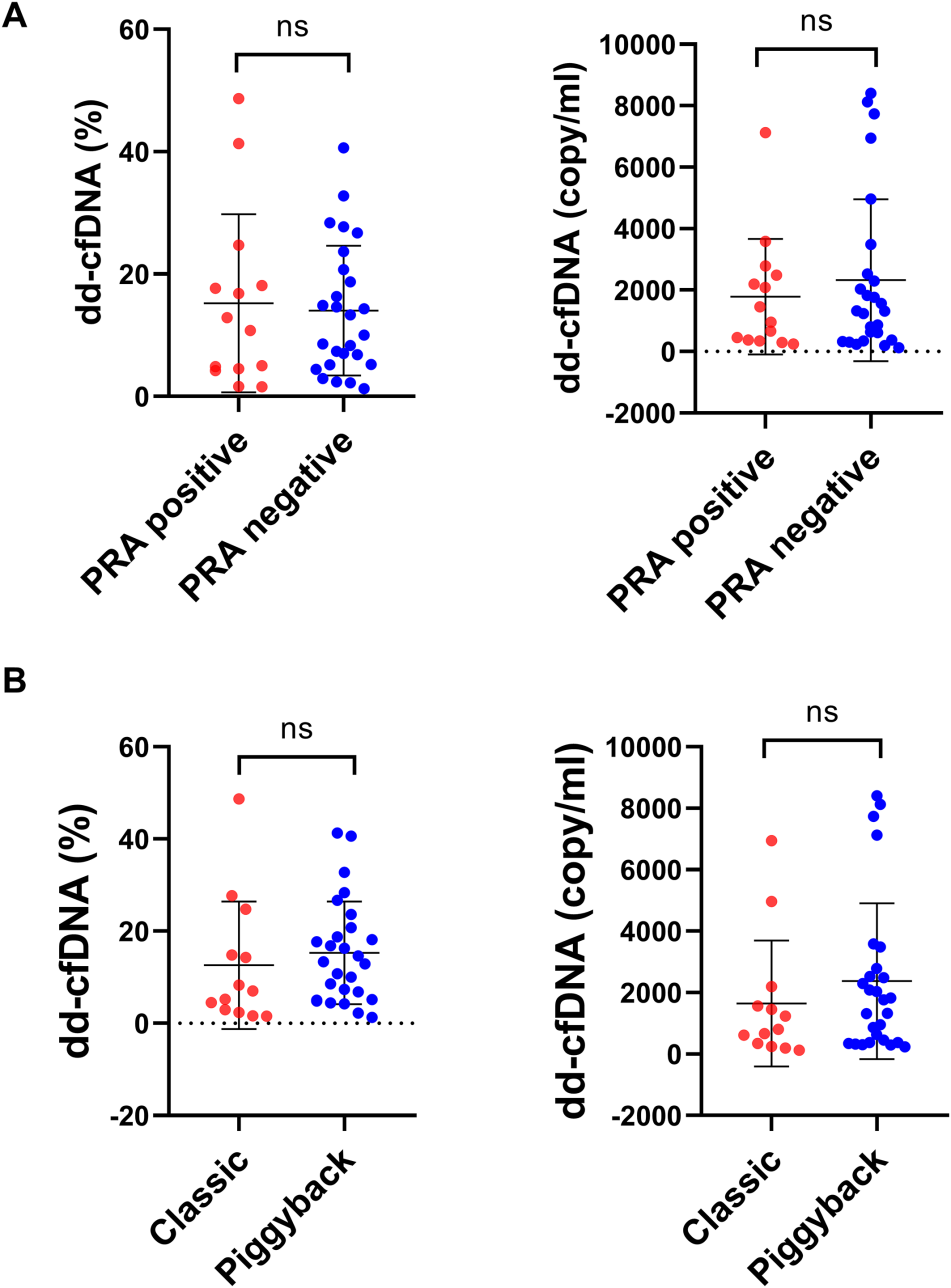
Subgroup analysis of dd-cfDNA levels. **(A)** Comparison of dd-cfDNA fraction (%) and absolute concentration (copies/mL) between patients with PRA-positive and PRA-negative status. **(B)** Comparison of dd-cfDNA levels between recipients undergoing classic and piggyback liver transplantation. No significant differences were observed between subgroups (*p* > 0.05). Data are presented as mean ± SD, and statistical analysis was performed using the Mann–Whitney *U* test.

To explore potential confounding factors, we further analyzed dd-cfDNA levels according to pretransplant panel reactive antibody (PRA) status and surgical technique (**Figure 5**). Neither dd-cfDNA% nor absolute dd-cfDNA differed significantly between PRA-positive and PRA-negative recipients, and no significant differences were observed between patients who underwent classic orthotopic transplantation versus the piggyback technique (all *p* > 0.05). These results indicate that dd-cfDNA-based detection of AR is largely independent of baseline immunologic sensitization and operative approach, supporting its robustness and generalizability as a noninvasive tool for graft surveillance.

### 3.5 Subgroup analyses of dd-cfDNA in different clinical settings

**Figure 6:**
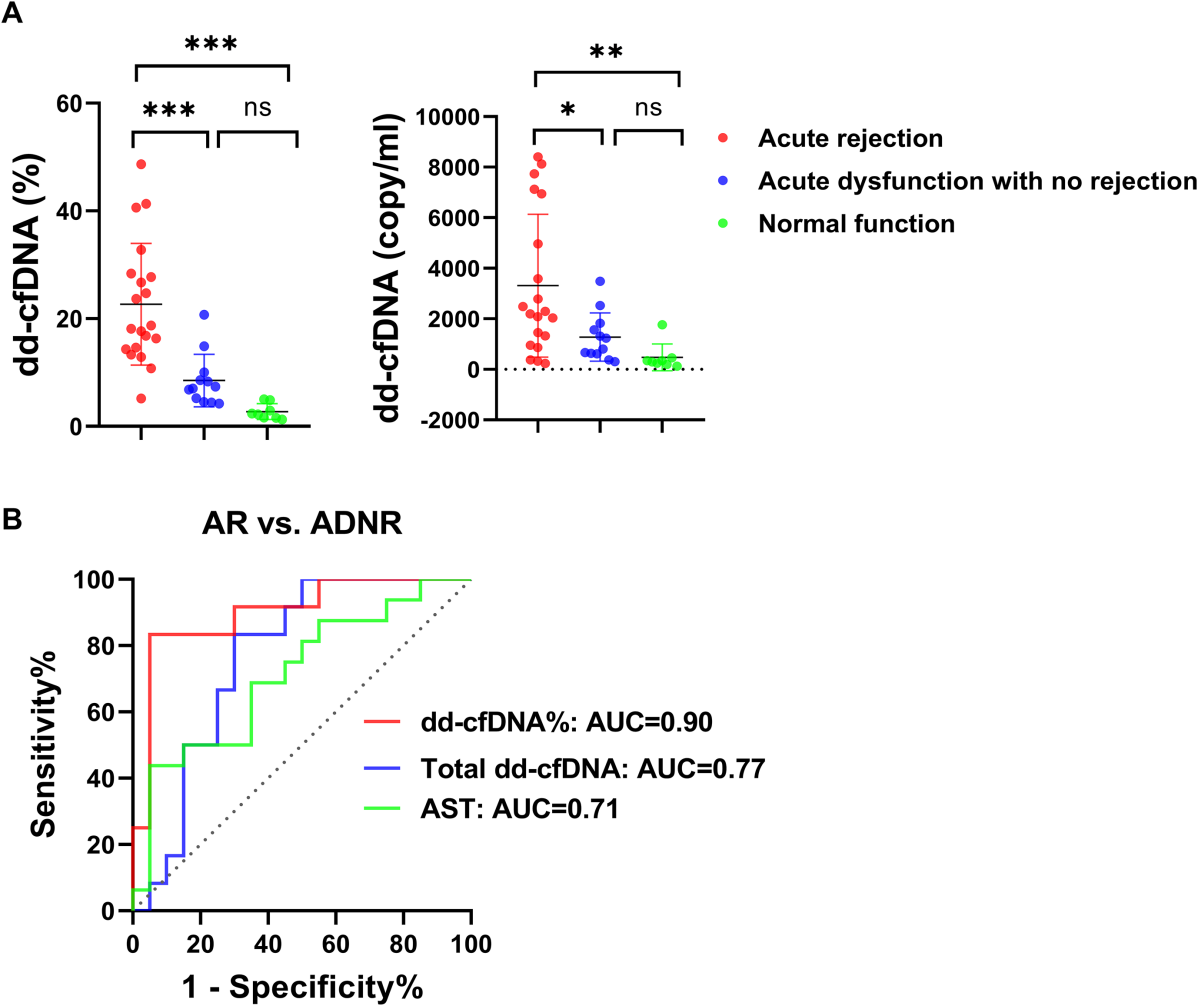
Subgroup analysis of dd-cfDNA levels and diagnostic performance for distinguishing acute rejection from acute dysfunction without rejection. **(A)** Comparison of fractional dd-cfDNA (%) and total dd-cfDNA (copies/mL) among AR, ADNR, and normal function groups. **(B)** ROC curves of fractional dd-cfDNA, total dd-cfDNA, and AST for distinguishing AR from ADNR. Statistical comparisons were performed using the Mann–Whitney U test. Levels of statistical significance are indicated as: *P < 0.05; **P < 0.01; ***P < 0.001; ns indicates no significant difference.

To further define the clinical context of the pooled analysis, we performed subgroup analyses in which recipients were categorized into acute rejection (AR), acute dysfunction with no rejection (ADNR), and normal function. Fractional dd-cfDNA (%) was significantly higher in the AR group than in both the ADNR group and the normal function group, whereas no significant difference was observed between the ADNR and normal function groups. Total dd-cfDNA was also significantly higher in the AR group than in the ADNR group and the normal function group; however, the degree of discrimination was less marked than that observed for fractional dd-cfDNA (**Figure 6A**). We then performed ROC analysis to evaluate the ability of dd-cfDNA to distinguish AR from ADNR. The AUCs were 0.90 for fractional dd-cfDNA, 0.77 for total dd-cfDNA, and 0.71 for AST. The optimal cutoff value for dd-cfDNA% was 15.58%, with a sensitivity of 91.7% and a specificity of 70.0%. In comparison, the optimal cutoff value for total dd-cfDNA was 2508 copies/mL, with a sensitivity of 83.3% and a specificity of 55.0% (**Figure 6B**). These findings suggest that fractional dd-cfDNA has better discriminatory performance than total dd-cfDNA for differentiating AR-related graft dysfunction from non-rejection-related acute dysfunction, suggesting potential value in helping identify which patients with graft dysfunction may warrant biopsy.

Within the protocol biopsy cohort, fractional dd-cfDNA (%) was significantly higher in recipients with subclinical acute rejection than in those with no rejection on surveillance biopsy (**Supplementary Figure 4**), whereas total dd-cfDNA did not differ significantly between the two groups, supporting the potential utility of dd-cfDNA as a surveillance biomarker for detecting subclinical rejection.

## 4 Discussion

Timely and accurate diagnosis of AR remains a critical challenge in LT. While liver biopsy remains the gold standard for diagnosing AR, it is invasive, expensive, and prone to sampling error (27–29). Therefore, identifying noninvasive biomarkers with high diagnostic accuracy is essential for improving patient outcomes. Our study evaluated the diagnostic performance of dd-cfDNA as a biomarker for detecting AR in liver transplant recipients. The results showed that dd-cfDNA levels were significantly elevated in patients with biopsy-confirmed AR compared to those without rejection. Although warm ischemia time was significantly longer in the AR group, correlation analysis did not show a significant association between WIT and either fractional or absolute dd-cfDNA in this cohort. In addition, all diagnostic samples were obtained from postoperative day 14 onward, after the early high-variability period associated with ischemia-reperfusion injury. Nevertheless, residual confounding related to ischemia-related graft injury cannot be completely excluded. Then two cutoff thresholds for dd-cfDNA were assessed: dd-cfDNA% ≥10.39% and dd-cfDNA ≥1928 copies/mL. The diagnostic performance of dd-cfDNA% ≥10.39% demonstrated a sensitivity of 95%, specificity of 90%, positive predictive value (PPV) of 90.5%, and negative predictive value (NPV) of 94.7%. In comparison, for dd-cfDNA ≥1928 copies/mL, sensitivity decreased to 70%, while specificity remained at 90%, with a PPV of 87.5% and an NPV of 75.0%. dd-cfDNA% (AUC = 0.940) exhibited superior diagnostic performance over both absolute dd-cfDNA concentration (copies/mL) and routine liver function tests, highlighting its excellent ability to identify AR. Furthermore, dd-cfDNA levels rapidly decreased following effective anti-rejection therapy, which was consistent with clinical and histological improvements, further supporting its dynamic value in the diagnosis and monitoring of AR.

Recent studies have firmly established dd-cfDNA as a promising biomarker for detecting acute rejection (AR) in liver transplant recipients (LTR). Our findings further support this, providing additional evidence for the utility of dd-cfDNA in liver transplant management. For instance, Levitsky et al. demonstrated that dd-cfDNA is highly effective in distinguishing AR from normal liver function, with an area under the curve (AUC) of 0.95 and a cutoff of 5.3% (30). Similarly, Kanamori et al. reported an even higher AUC of 0.975 for AR detection (31). In a pediatric cohort, Zhao et al. found that elevated dd-cfDNA% (cutoff = 28.7%) was associated with rejection, outperforming conventional liver function tests in diagnostic accuracy (32). Additionally, Fernández-Galán et al. identified a cutoff of 13.8% as clinically significant in adult liver transplant recipients (33). In line with these studies, our research shows that dd-cfDNA levels are significantly elevated in LTR experiencing AR, and they decrease following successful treatment.

In our study, the cutoffs for AR detection were set at 10.39% for fractional dd-cfDNA and 1928 copies/mL for absolute dd-cfDNA, yielding a sensitivity of 95% and specificity of 90%. However, these cutoff values vary significantly between studies, ranging from 5% to 30%. This variability is likely due to differences in assay methods (e.g., ddPCR, STR-based PCR, SNP-NGS), patient populations (e.g., adult vs. pediatric, different ethnic backgrounds), and the clinical context in which dd-cfDNA is measured (e.g., early vs. late post-transplant, biopsy-confirmed vs. clinically suspected rejection) (34). These disparities highlight the lack of a standardized dd-cfDNA threshold for clinical use in liver transplantation, emphasizing the need for further multi-center studies to validate optimal cutoff values across diverse populations. Therefore, rather than defining a universal cutoff, our study provides additional cohort-specific evidence regarding the diagnostic performance of dd-cfDNA and contributes to the growing literature on threshold variability across liver transplant studies.

The dd-cfDNA has emerged as a robust and noninvasive biomarker for detecting allograft injury and acute rejection across multiple solid organ transplants, including kidney, heart, and lung. In renal transplant, dd-cfDNA exhibits high sensitivity and specificity in detecting acute rejection. Mantios et al. identified an optimal threshold of 0.5% (AUC 0.804), surpassing creatinine in discriminating rejection. Additionally, dd-cfDNA concentrations decreased notably post successful anti-rejection intervention, indicating its utility in treatment monitoring and long-term graft function prognosis (35). In heart transplantation, Böhmer et al. illustrated that both absolute dd-cfDNA concentration and donor fraction (DF) could effectively differentiate rejection from quiescence, with absolute quantification (threshold: 25 copies/mL, AUC 0.87) outperforming DF (AUC 0.75), particularly in cases of symptomatic rejection. This finding highlights the clinical utility of dd-cfDNA as a complement or alternative to endomyocardial biopsy (36). In lung transplantation, several prospective studies have indicated that a plasma dd-cfDNA fraction of approximately 1% can distinguish acute rejection and chronic lung allograft dysfunction from stable grafts, with areas under the receiver operating characteristic curve (AUCs) ranging from approximately 0.87 to 0.90, sensitivities between 77% and 100%, and specificities between 73% and 84% (37). Collectively, these studies support dd-cfDNA as a universal, dynamic biomarker reflecting graft injury across organ systems. It not only aids in early and accurate detection of acute rejection but also tracks therapeutic efficacy and long-term graft stability, representing a paradigm shift toward precision and minimally invasive transplant monitoring.

Beyond dd-cfDNA alone, combining dd-cfDNA with other molecular biomarkers may represent a promising strategy to further improve diagnostic precision in transplantation. Previous studies have explored the potential value of integrating dd-cfDNA with biomarkers such as CXCL-10, gene expression profiling, and microRNAs (38–40). However, since no additional biomarkers or integrative analyses were assessed in the present study, this concept should be considered a future research direction. Further prospective studies are warranted to determine whether such multimarker approaches provide added value beyond dd-cfDNA alone in liver transplantation.

Our study has several limitations. It was conducted at a single center with a limited sample size, which may restrict generalizability. In addition, although recipients from the for-cause and surveillance settings were pooled according to biopsy outcome for the primary analysis, more detailed subgroup analyses were limited by the sample size. Moreover, while dd-cfDNA showed strong diagnostic performance in our cohort, its levels may still be influenced by confounding factors such as infection and ischemia–reperfusion injury. Future multicenter studies with larger cohorts and diverse causes of graft dysfunction are needed to validate diagnostic thresholds, perform clinically relevant subgroup analyses, and evaluate longitudinal dd-cfDNA kinetics.

## 5 Conclusions

In conclusion, dd-cfDNA is a promising noninvasive biomarker for the diagnosis and monitoring of acute rejection in liver transplant recipients. Despite variability in cutoff values across studies, our results demonstrate that dd-cfDNA can reliably identify acute rejection with high diagnostic accuracy in this cohort. Further large-scale, multicenter prospective studies are needed to refine cutoff values, validate its clinical utility in routine monitoring, and determine whether dd-cfDNA-guided strategies may enable earlier detection of rejection and reduce reliance on invasive diagnostic procedures.

## Data Availability

The original contributions presented in the study are included in the article/**Supplementary Material**. Further inquiries can be directed to the corresponding author/s.

## References

[1] Xu X . State of the art and perspectives in liver transplantation. Hepatobiliary Pancreat Dis Int. (2023) 22:1–3. doi: 10.1016/j.hbpd.2022.12.001. PMID: 36528548

[2] Sugawara Y Hibi T . Recent trends and new developments in liver transplantation. Biosci Trends. (2024) 18:206–11. doi: 10.5582/bst.2024.01176. PMID: 38945855

[3] Feng S Roll GR Rouhani FJ Sanchez Fueyo A . The future of liver transplantation. Hepatology. (2024) 80:674–97. doi: 10.1097/hep.0000000000000873. PMID: 38537154

[4] Ashwat E Spitz FJ Abdullah A Elias C Mail-Anthony J Subedi S . Survival outcomes of liver transplantation amid rising recipient and donor risk profiles. Clin Transplant. (2025) 39:e70349. doi: 10.1111/ctr.70349. PMID: 41137651 PMC12553133

[5] Levitsky J Goldberg D Smith AR Mansfield SA Gillespie BW Merion RM . Acute rejection increases risk of graft failure and death in recent liver transplant recipients. Clin Gastroenterol Hepatol. (2017) 15:584–593.e2. doi: 10.1016/j.cgh.2016.07.035. PMID: 27567694 PMC5326609

[6] Kohut TJ Barandiaran JF Keating BJ . Genomics and liver transplantation: Genomic biomarkers for the diagnosis of acute cellular rejection. Liver Transpl. (2020) 26:1337–50. doi: 10.1002/lt.25812. PMID: 32506790

[7] Montano-Loza AJ Rodríguez-Perálvarez ML Pageaux G-P Sanchez-Fueyo A Feng S . Liver transplantation immunology: Immunosuppression, rejection, and immunomodulation. J Hepatol. (2023) 78:1199–215. doi: 10.1016/j.jhep.2023.01.030. PMID: 37208106

[8] Dai H Zheng Y Thomson AW Rogers NM . Transplant tolerance induction: Insights from the liver. Front Immunol. (2020) 11:1044. doi: 10.3389/fimmu.2020.01044. PMID: 32582167 PMC7289953

[9] Rastogi A . Liver transplant biopsy interpretation: Diagnostic considerations and conundrums. Indian J Pathol Microbiol. (2022) 65:245–57. doi: 10.4103/ijpm.ijpm_1090_21. PMID: 35435355

[10] Bardhi E McDaniels J Rousselle T Maluf DG Mas VR . Nucleic acid biomarkers to assess graft injury after liver transplantation. JHEP Rep. (2022) 4:100439. doi: 10.1016/j.jhepr.2022.100439. PMID: 35243279 PMC8856989

[11] Lim DZ Low N Jackett L Ma R Jones R Testro A . Clinical and financial impacts of abnormal liver biochemistry after liver transplantation. BMC Res Notes. (2023) 16:7. doi: 10.1186/s13104-022-06268-w. PMID: 36707903 PMC9883895

[12] Knight SR Thorne A Lo Faro ML . Donor-specific cell-free DNA as a biomarker in solid organ transplantation. A systematic review. Transplantation. (2019) 103:273–83. doi: 10.1097/tp.0000000000002482. PMID: 30308576

[13] Pan Q Zhou AW Wang BR Xiao WL Gao YM Liu HY . Diagnostic and predictive biomarkers of acute rejection after liver transplantation. Int J Surg. (2025) 111:3908–19. doi: 10.1097/js9.0000000000002358. PMID: 40505038 PMC12165572

[14] Edwards RL Menteer JD Lestz RM Baxter-Lowe LA . Cell-free DNA as a solid-organ transplant biomarker: Technologies and approaches. biomark Med. (2022) 16:401–15. doi: 10.2217/bmm-2021-0968. PMID: 35195028

[15] Tsao DS . Absolute quantification of cell-free DNA for prenatal genetics and oncology. Trends Biotechnol. (2025) 43:732–3. doi: 10.1016/j.tibtech.2024.12.003. PMID: 39794210

[16] Schütz E Fischer A Beck J Harden M Koch M Wuensch T . Graft-derived cell-free DNA, a noninvasive early rejection and graft damage marker in liver transplantation: A prospective, observational, multicenter cohort study. PloS Med. (2017) 14:e1002286. doi: 10.1371/journal.pmed.1002286 28441386 PMC5404754

[17] Pettersson L Westerling S Talla V Sendel A Wennberg L Olsson R . Development and performance of a next generation sequencing (NGS) assay for monitoring of dd-cfDNA post solid organ transplantation. Clin Chim Acta. (2024) 552:117647. doi: 10.1016/j.cca.2023.117647. PMID: 37951377

[18] Clausen FB Jørgensen KL Wardil LW Nielsen LK Krog GR . Droplet digital PCR-based testing for donor-derived cell-free DNA in transplanted patients as noninvasive marker of allograft health: Methodological aspects. PloS One. (2023) 18:e0282332. doi: 10.1371/journal.pone.0282332. PMID: 36827438 PMC9955980

[19] Oellerich M Sherwood K Keown P Schütz E Beck J Stegbauer J . Liquid biopsies: Donor-derived cell-free DNA for the detection of kidney allograft injury. Nat Rev Nephrol. (2021) 17:591–603. doi: 10.1038/s41581-021-00428-0. PMID: 34031575

[20] Oellerich M Budde K Osmanodja B Bornemann-Kolatzki K Beck J Schütz E . Donor-derived cell-free DNA as a diagnostic tool in transplantation. Front Genet. (2022) 13:1031894. doi: 10.3389/fgene.2022.1031894. PMID: 36339004 PMC9634115

[21] Jiménez-Coll V El Kaaoui El Band J Llorente S González-López R Fernández-González M Martínez-Banaclocha H . All that glitters in cfdna analysis is not gold or its utility is completely established due to graft damage: A critical review in the field of transplantation. Diagnostics (Basel). (2023) 13:1982. doi: 10.3390/diagnostics13121982. PMID: 37370877 PMC10297394

[22] Aubert O Ursule-Dufait C Brousse R Gueguen J Racapé M Raynaud M . Cell-free DNA for the detection of kidney allograft rejection. Nat Med. (2024) 30:2320–7. doi: 10.1038/s41591-024-03087-3. PMID: 38824959 PMC11333280

[23] Khachatoorian Y KhaChadourian V Chang E Sernas ER Reed EF Deng M . Noninvasive biomarkers for prediction and diagnosis of heart transplantation rejection. Transplant Rev (Orlando). (2021) 35:100590. doi: 10.1016/j.trre.2020.100590. PMID: 33401139

[24] Sorbini M Togliatto G Mioli F Simonato E Marro M Cappuccio M . Validation of a simple, rapid, and cost-effective method for acute rejection monitoring in lung transplant recipients. Transpl Int. (2022) 35:10546. doi: 10.3389/ti.2022.10546. PMID: 35755857 PMC9221674

[25] Cuervo Florez M Bruner J Zarrinpar A . Progress and challenges in diagnosis and treatment of rejection following liver transplantation. Curr Opin Organ Transplant. (2021) 26:669–74. doi: 10.1097/mot.0000000000000924. PMID: 34581291

[26] Demetris AJ Bellamy C Hübscher SG O'Leary J Randhawa PS Feng S . 2016 comprehensive update of the Banff Working Group on liver allograft pathology: Introduction of antibody-mediated rejection. Am J Transplant. (2016) 16:2816–35. doi: 10.1111/ajt.13909. PMID: 27273869

[27] Lee BT Fiel MI Schiano TD . Antibody-mediated rejection of the liver allograft: An update and a clinico-pathological perspective. J Hepatol. (2021) 75:1203–16. doi: 10.1016/j.jhep.2021.07.027. PMID: 34343613

[28] Kamali K Schmelzle M Kamali C Brunnbauer P Splith K Leder A . Sensing acute cellular rejection in liver transplant patients using liver-derived extracellular particles: A prospective, observational study. Front Immunol. (2021) 12:647900. doi: 10.3389/fimmu.2021.647900. PMID: 34025656 PMC8131523

[29] Kumar S Mohapatra N Borle DP Choudhury A Sarin S Gupta E . Non invasive diagnosis of acute cellular rejection after liver transplantation - Current opinion. Transpl Immunol. (2018) 47:1–9. doi: 10.1016/j.trim.2018.02.002. PMID: 29452168

[30] Levitsky J Kandpal M Guo K Kleiboeker S Sinha R Abecassis M . Donor-derived cell-free DNA levels predict graft injury in liver transplant recipients. Am J Transplant. (2022) 22:532–40. doi: 10.1111/ajt.16835. PMID: 34510731

[31] Kanamori H Yamada Y Ito Y Shirosaki K Yamagishi S Maeda Y . Noninvasive graft monitoring using donor-derived cell-free DNA in Japanese liver transplantation. Hepatol Res. (2024) 54:300–14. doi: 10.1111/hepr.13978. PMID: 37850337

[32] Zhao D Zhou T Luo Y Wu C Xu D Zhong C . Preliminary clinical experience applying donor-derived cell-free DNA to discern rejection in pediatric liver transplant recipients. Sci Rep. (2021) 11:1138. doi: 10.1038/s41598-020-80845-6. PMID: 33441886 PMC7807012

[33] Fernández-Galán E Badenas C Fondevila C Jiménez W Navasa M Puig-Butillé JA . Monitoring of donor-derived cell-free DNA by short tandem repeats: Concentration of total cell-free DNA and fragment size for acute rejection risk assessment in liver transplantation. Liver Transpl. (2022) 28:257–68. doi: 10.1002/lt.26272 34407295

[34] Zhong Y Hu X Li X Qiao Y Li H Zhou S . Advances and challenges in the application of donor-derived cell-free DNA for diagnosis and treatment in liver transplantation: A narrative review. BMC Surg. (2025) 25:203. doi: 10.1186/s12893-025-02911-y. PMID: 40361085 PMC12070513

[35] Mantios E Filiopoulos V Constantoulakis P Liapis G Vittoraki A Casas S . Assessment of donor derived cell free DNA (dd-cfDNA) at surveillance and at clinical suspicion of acute rejection in renal transplantation. Transpl Int. (2023) 36:11507. doi: 10.3389/ti.2023.11507. PMID: 37901296 PMC10603235

[36] Böhmer J Wåhlander H Tran-Lundmark K Odermarsky M Sjöborg Alpman M Asp J . Absolute quantification of donor-derived cell-free DNA following pediatric and adult heart transplantation. J Heart Lung Transplant. (2025) 44:1638–47. doi: 10.1016/j.healun.2025.04.024 40345563

[37] Keller M Agbor-Enoh S . Donor-derived cell-free DNA for acute rejection monitoring in heart and lung transplantation. Curr Transplant Rep. (2021) 8:351–8. doi: 10.1007/s40472-021-00349-8. PMID: 34754720 PMC8570240

[38] Yu Q Zhang Y Gao Z Wang B Yang H He L . Diagnostic value of dd-cfDNA and CXCL-10 in kidney allograft recipients for identifying acute rejection. Transpl Immunol. (2025) 93:102307. doi: 10.1016/j.trim.2025.102307. PMID: 41057101

[39] Gondi KT Kao A Linard J Austin BA Everley MP Fendler TJ . Single-center utilization of donor-derived cell-free DNA testing in the management of heart transplant patients. Clin Transplant. (2021) 35:e14258. doi: 10.1111/ctr.14258. PMID: 33606316

[40] Julian J Millán O Titos E Ruiz P Fundora Y Díaz A . Donor-derived cell-free DNA and miRNA monitoring for the early prediction and diagnosis of liver allograft rejection and patient outcomes. Front Immunol. (2025) 16:1604200. doi: 10.3389/fimmu.2025.1604200. PMID: 40630960 PMC12234302

