# Supplementary material for: Donor-derived cell-free DNA as a noninvasive biomarker for diagnosis and monitoring of acute rejection after liver transplantation
Source: Front Immunol. 2026 May 11;17:1769538. doi: 10.3389/fimmu.2026.1769538 (PMC13199101; doi:10.3389/fimmu.2026.1769538)
Supplement: Supplementary file 1 [file Table1.doc]

**Donor-derived cell-free DNA as a noninvasive biomarker for diagnosis and monitoring of acute rejection after liver transplantation**

**Author name:**

Zhigao Deng^1, *^, Meicheng yang^1, *^, Quanwei Cheng ^1^, Zhongshan Lu ^1^, Qifa Ye^1, 2^, Yan Xiong ^1, #^, Shaojun Ye ^1, #^

**Supplemental Materials**

**Supplementary Fig. 1**

**
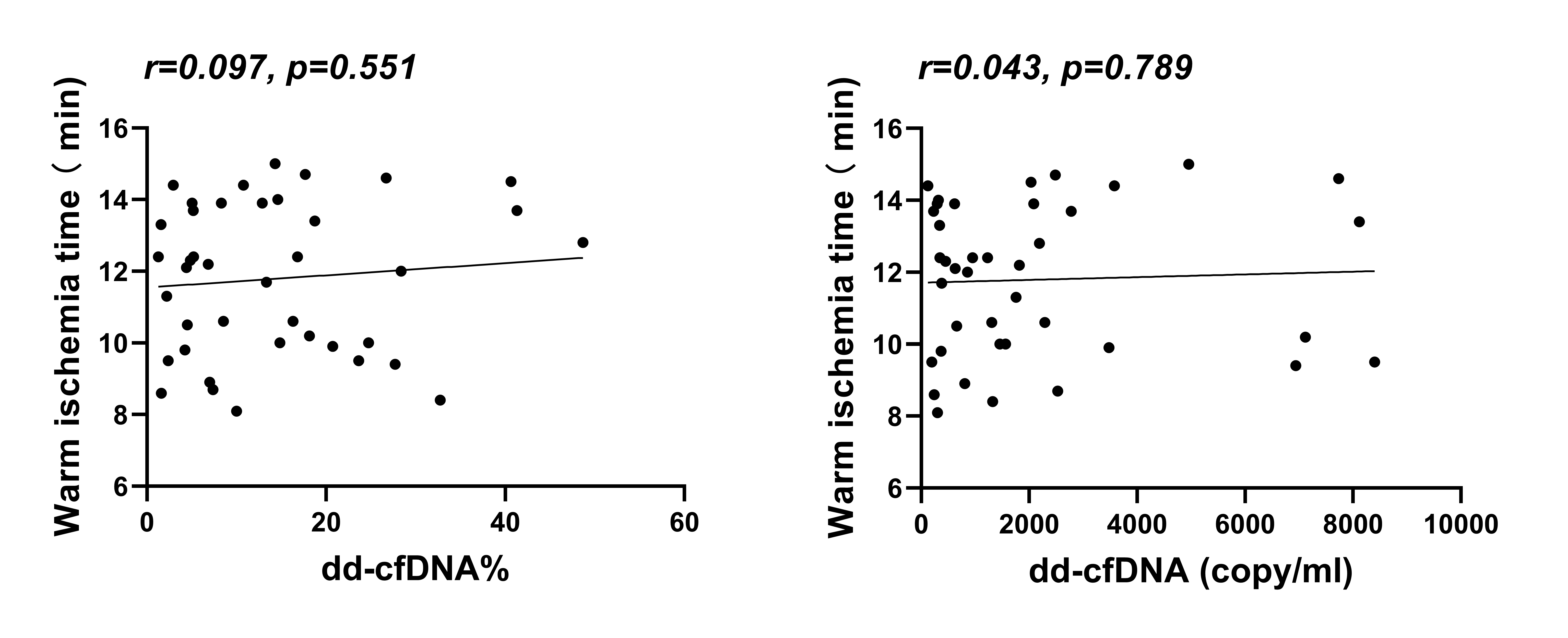
**

**Supplementary Fig. 1. Correlation of warm ischemia time with fractional and absolute dd-cfDNA levels.** Scatter plots showing the relationship between warm ischemia time and fractional dd-cfDNA (%) or absolute dd-cfDNA (copies/mL). Correlation analysis was performed using Spearman’s rank correlation test. Correlation coefficients and corresponding p values are shown in each panel.

**Supplementary Fig. 2**

**
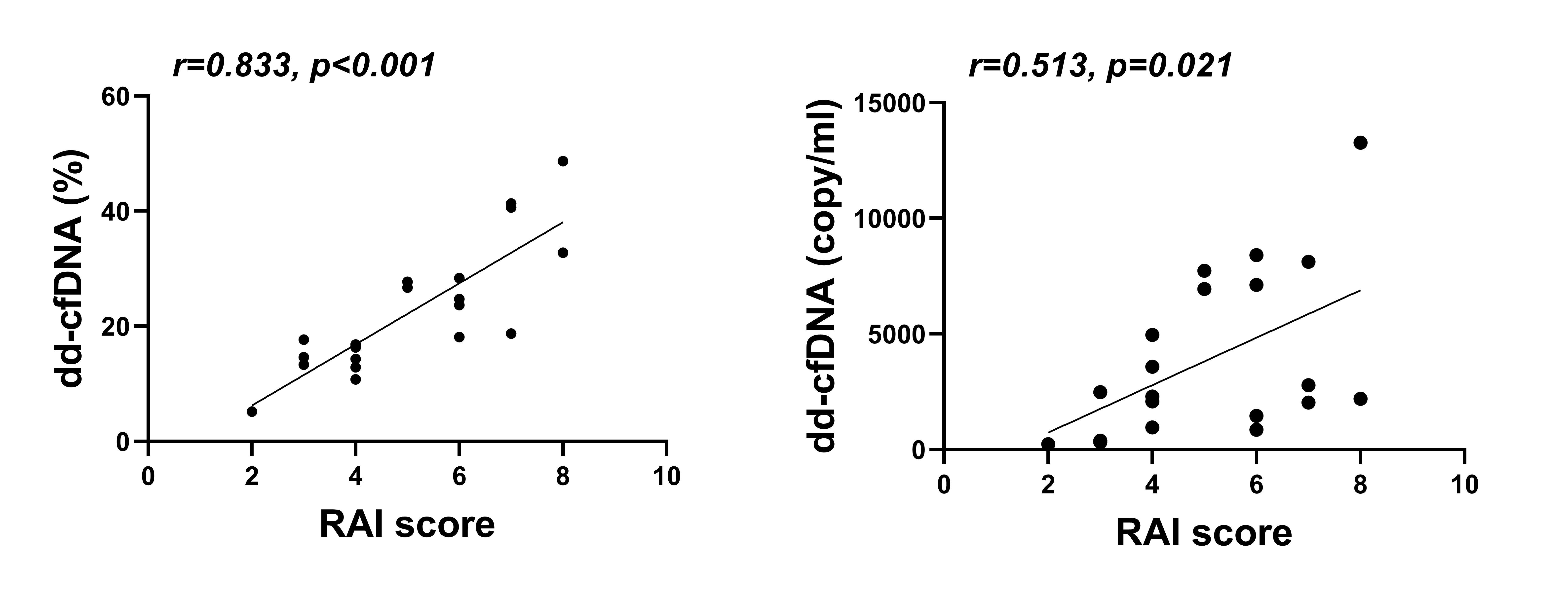
**

**Supplementary Fig. 2. Correlation between dd-cfDNA levels and Banff rejection activity index (RAI) score.** Scatter plots showing the correlations between fractional dd-cfDNA (%) and RAI score (left), and between total dd-cfDNA (copies/mL) and RAI score (right) in recipients with acute rejection. Correlation analysis was performed using Spearman’s rank correlation test. Correlation coefficients and corresponding p values are shown in each panel.

**Supplementary Fig. 3**


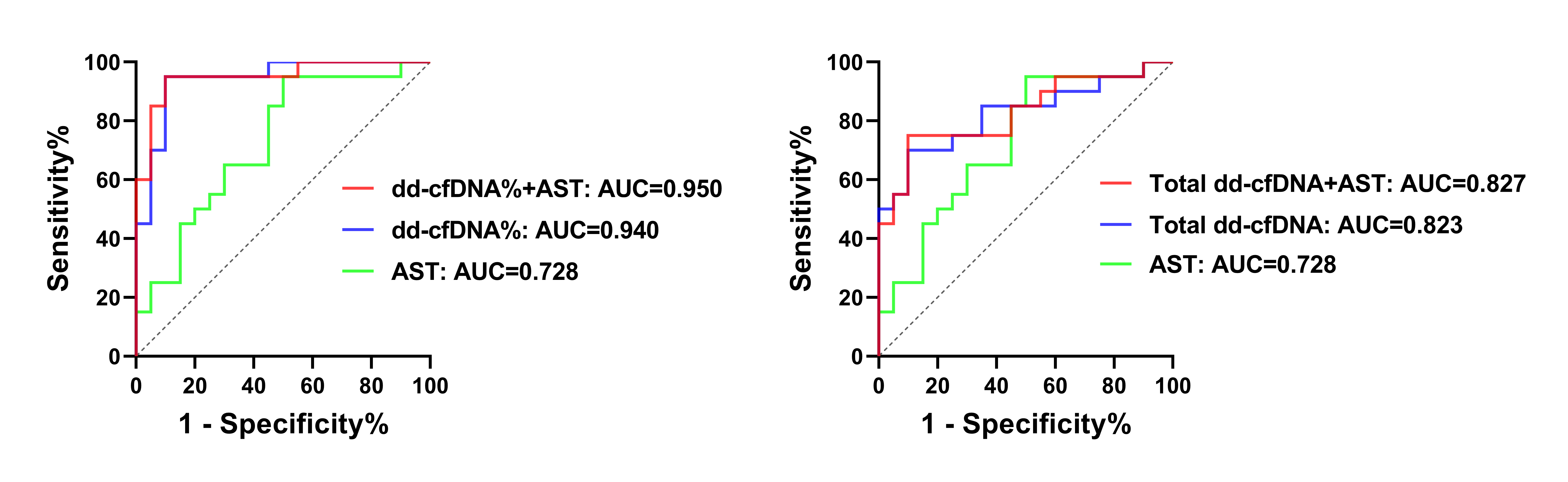


**Supplementary Fig. 3. Combined ROC analysis of dd-cfDNA and AST for diagnosing acute rejection.** Receiver operating characteristic (ROC) curves comparing combined models of fractional dd-cfDNA + AST and total dd-cfDNA + AST with the corresponding single markers, including fractional dd-cfDNA alone, total dd-cfDNA alone, and AST alone, for diagnosing acute rejection. The AUCs are shown in each panel.

.

**Supplementary Fig. 4**

**
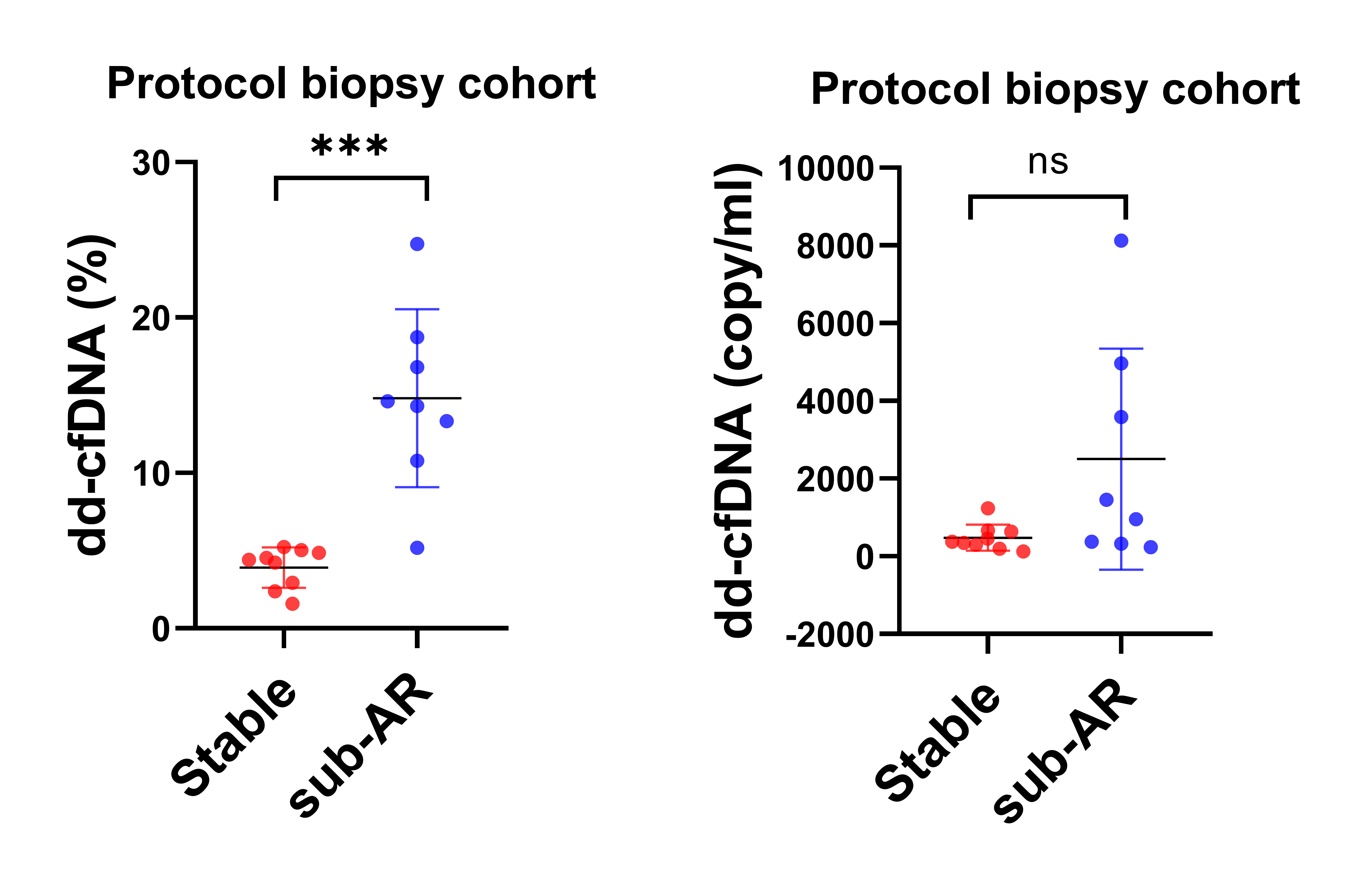
**

**Supplementary Fig. 4. dd-cfDNA levels in the protocol biopsy cohort.** Comparison of fractional dd-cfDNA (%) and total dd-cfDNA (copies/mL) between recipients with stable graft function and those with subclinical acute rejection (sub-AR) in the protocol biopsy cohort. Statistical comparisons were performed using the Mann–Whitney U test. Levels of statistical significance are indicated as: ***, *P* < 0.001; ns indicates no significant difference.
